# Analytical Quality-by-Design-Based Development of an ELISA for *In Vitro* Assessment of Human Tetanus Immunoglobulin Potency

**DOI:** 10.4014/jmb.2603.03028

**Published:** 2026-07-01

**Authors:** Minjung Son, Boksik Cha, Yunkyung Na, Hyungjung Koh, Chan Woong Choi, Jangwook Lee, Sung-Jin Yoon, Young-Joo Jeon

**Affiliations:** 1 Metabolic Regulation Research Center, Korea Research Institute of Bioscience and Biotechnology (KRIBB), Daejeon 34141, Republic of Korea; 2 Department of Functional Genomics, KRIBB School of Bioscience, University of Science and Technology (UST), Daejeon, Republic of Korea; 3 Department of Physiology, Daegu Catholic University School of Medicine, Daegu, Republic of Korea; 4 Disease Target Structure Research Center, Korea Research Institute of Bioscience and Biotechnology (KRIBB), Daejeon 34141, Republic of Korea; 5 Blood Products Division, Biopharmaceutical and Herbal Medicine Evaluation Department, National Institute of Food and Drug Safety Evaluation, Republic of Korea; 6 Biotherapeutics Translational Research Center, Korea Research Institute of Bioscience and Biotechnology (KRIBB), Daejeon 34141, Republic of Korea; 7 Department of Biomolecular Science, KRIBB School of Bioscience, University of Science and Technology (UST), Daejeon, Republic of Korea; 8 Environmental Disease Research Center, Korea Research Institute of Bioscience and Biotechnology (KRIBB), Daejeon 34141, Republic of Korea

**Keywords:** Tetanus immunoglobulin, Enzyme-linked immunosorbent assay, Analytical quality-by-design, Method validation, *In vitro* potency

## Abstract

An enzyme-linked immunosorbent assay (ELISA) method for the *in vitro* evaluation of tetanus immunoglobulin potency was developed and validated using analytical quality-by-design principles, and a quality risk assessment identified critical method parameters, including antigen coating time, washing, color development time, and antibody incubation time and temperature. These variables were systematically optimized using Design of Experiments within JMP software. Following this, the concentrations of tetanus antigen and anti-tetanus antibody were further optimized to enhance the assay performance. The method demonstrated excellent linearity (R^2^ > 0.99), accuracy, precision, and recovery in accordance with the International Council for Harmonization Q2(R1) and United States Pharmacopeia <1033> guidelines. A comparative analysis using two commercial ELISA kits revealed no statistically significant differences. These findings support the use of an analytical quality-by-design-based ELISA as a scientifically robust, reproducible, and ethical method suitable for *in vitro* evaluation of tetanus immunoglobulin potency in alignment with regulatory expectations.

## Introduction

Tetanus is a potentially fatal disease caused by *Clostridium tetani*, which produces neurotoxins that lead to muscle spasms and respiratory failure. Despite effective vaccination programs, infections continue to occur, particularly in individuals with incomplete immunization or in resource-limited settings [1]. Tetanus immunoglobulin (TIG) is an essential biological product used for passive immunization of individuals exposed to *C. tetani*, particularly in those with incomplete vaccination histories or contaminated wounds [2, 3]. Given that TIG is derived from human plasma, it must undergo rigorous quality control testing to ensure potency, safety, and batch-to-batch consistency prior to clinical use. Among these quality attributes, potency is especially critical as it relates directly to the therapeutic efficacy of the product [4].

Traditionally, the potency of TIG has been evaluated using the tetanus neutralization test (TNT), an *in vivo* bioassay involving the administration of tetanus toxin and antitoxin to mice to observe survival outcomes [5]. Despite its longstanding use, TNT has several major limitations, including ethical concerns regarding animal welfare, low throughput, high inter-animal variability, and long assay duration [6]. Thus, the need for validated *in vitro* alternatives that are faster, more reproducible, and more ethically justified has become increasingly apparent. Global regulatory agencies, including the World Health Organization, have actively encouraged the development and adoption of scientifically justified animal-free testing methods [7]. These initiatives are further supported by the internationally recognized 3Rs principle: Replacement, Reduction, and Refinement, which advocates minimizing animal use and suffering, while ensuring scientific validity [8, 9].

The concept of quality-by-design was first introduced by the U.S. Food and Drug Administration in 2004 to improve the robustness and consistency of pharmaceutical manufacturing [10]. This approach was later extended to analytical method development, leading to the establishment of analytical quality-by-design (AQbD) [11]. The United States Pharmacopeia (USP) supported AQbD implementation by publishing chapters <1032> on biological assay development (USP 2020a), <1033> on assay validation (USP 2020b), and <1210> on statistical tools for procedure validation (USP, 2020c) [12-14]. More recently, the USP <1220> (USP 2022) [15] and International Council for Harmonization (ICH) Q14 and Q2(R^2^) guidelines provided updated frameworks for the analytical procedure lifecycle.

AQbD has been increasingly used in the development of bioanalytical methods. AQbD offers a systematic framework combining risk assessment, a process used to identify and prioritize variables that may impact method performance, often conducted using tools such as Failure Mode and Effects Analysis (FMEA). The framework is also combined with the Design of Experiments (DoE) strategy and statistical modeling to identify and control critical method parameters (CMPs), resulting in a robust and well-characterized analytical method [16, 17]. This approach enhances the assay performance while supporting lifecycle management and regulatory acceptance.

In the present study, we applied AQbD principles to develop and validate an enzyme-linked immunosorbent assay (ELISA)-based *in vitro* potency assay for TIG detection. A comprehensive risk assessment was conducted to identify the critical assay parameters, including antigen coating time, incubation temperature and duration, washing conditions, and antibody concentration. Optimization was performed using DoE within JMP^®^ software, enabling multiple response analysis. The finalized method was validated according to the ICH and USP guidelines, and its performance was compared with two commercial ELISA kits to assess its equivalency and real-world applicability. This study offers a scientifically sound and ethically favorable alternative to TNT, contributing to the advancement of modern analytical strategies for the quality control of biologics. In contrast to previously reported ELISA-based assays and AQbD applications, this study focuses on the development of a fully AQbD-driven *in vitro* potency assay specifically for human TIG that is designed to meet current regulatory expectations for biological potency testing. By integrating a complete AQbD workflow—from Ishikawa risk assessment and FMEA-based factor prioritization to DoE-driven optimization, MODR definition, and guideline-based validation—this work provides a practical and transferable template for establishing animal-free potency assays for plasma-derived immunoglobulin products.

## Materials and Methods

### Materials

Tetanus toxin (MFDS-B-20-001, 156 L+/vial) was obtained from the Korean Ministry of Food and Drug Safety. The anti-tetanus human immunoglobulin used in this study included the Korean national reference standard (12/040, 32.74 IU/vial) and the European Pharmacopoeia reference standard provided by the European Directorate for the Quality of Medicines & HealthCare (EDQM, H111000, 45 IU/vial, France). A 0.05 M carbonate buffer (A 0.05 M carbonate buffer (Sigma, C3041, USA) was used to coat the plate, and phosphate-buffered saline (PBS) containing 0.05% Tween 20 (PBS-T) was used as a washing buffer. Tetanus toxin was diluted in carbonate buffer, and anti-TIG was diluted in PBS. The substrate for the ELISA assay was 3,3’,5,5’-Tetramethylbenzidine (TMB, Sigma, T8665, USA). A 0.5 M sulfuric acid (Merck, 1.09072.1000, Germany) was used as a stop solution. For comparison with commercial ELISAs, the SERION ELISA classic Tetanus IgG kit (SERION, ESR108G, Germany) and the VaccZyme Human Anti-tetanus Toxoid IgG EIA Kit (Binding Site, MK010.U, United Kingdom) were used.

### Analytical Target Profile (ATP)

The ATP was defined based on the ELISA experimental results, including critical parameters such as accuracy, precision, and recovery. AQbD principles were applied throughout the ELISA development to ensure that the method met all necessary quality requirements.

### National Reference Standard (NRS)

The Korean NRS for anti-tetanus human immunoglobulins (code 12/040) with a potency of 32.74 IU (International Unit) per vial was used in this study. The NRS is stored and managed by the National Institute of Food and Drug Safety Evaluation.

### Experimental Design and DoE Optimization

DoE is an essential components of the AQbD approach [18]. In the early phase of method development, a quality risk assessment was conducted to identify the key factors affecting ELISA performance. The primary factors influencing the ELISA outcomes were evaluated, including incubation time (for antigen, antibody, and color development), temperature (for antibody incubation and color development), and washing conditions. In addition, the concentrations of tetanus toxin and anti-TIG were optimized using an analytical design strategy.

For ELISA optimization, a diluted tetanus solution was dispensed into a 96-well microplate (0.0195-0.156 L+ per well) and incubated at 4°C for 12 h. After incubation, the wells were washed three times with PBS-T, followed by the addition of 200 μL blocking buffer, and incubated at 37°C for 1 h. Following another wash step, 1-4 mIU of anti-TIG per well (100 µL) was added to each well and incubated for 2 h at room temperature. Subsequently, 100 μL TMB substrate was added, and color development was performed for 5 min in the dark. The reaction was stopped by adding 100 μL of stop solution, and absorbance was measured at 450/630 nm using a microplate reader.

Critical method attributes and analytical procedure parameters were incorporated based on custom experimental designs using JMP^®^ software (version 18; SAS Institute, USA). The experimental Method Operable Design Region (MODR) was determined using the predictive profiler function in JMP 18^®^ software, allowing for the evaluation of method robustness.

### Signal-to-Noise Ratio (S/N)

The S/N was used to evaluate the assay performance and optimize the experimental conditions. This value was calculated by dividing the mean absorbance of the analyte by the standard deviation of the background signal. JMP 18^®^ software was used to identify the experimental conditions that yielded the highest S/N ratio, indicating improved assay sensitivity and precision.

### ELISA Validation

To validate the ELISA method developed, serial dilutions of NRS, ranging from 3274 to 0.5 mIU/mL, were used. The mean absorbance values were plotted against the logarithmic concentrations of NRS to construct a standard curve, which was subsequently fitted to a four-parameter logistic (4PL) model using GraphPad Prism version 10 (USA). The linear range was determined from the sigmoidal curve by identifying the concentration range in which optical density (OD) values formed a linear segment. A linear regression curve (y=mx+c) was applied to this segment to confirm linearity.

The accuracy and precision were evaluated under intermediate precision conditions by assessing the impact of analysts and analysis days. Two analysts performed the assay twice daily for two consecutive days, with each concentration level tested in at least three replicates per run (n ≥ 3), using different assay runs performed on different days and batches for all validation studies. Accuracy was calculated based on percent bias and its confidence interval, whereas precision was assessed using the geometric mean (GM), coefficient of variation (CV), and geometric CV (GCV). The optimized conditions identified from the DoE were fixed and evaluated under these validation settings, in independent experiments separate from the original DoE runs, demonstrating consistent performance across the independent experiments. All the values were within the pre-defined acceptance criteria outlined for ATP.

### Specificity Assessment

Specificity was assessed by performing spiked recovery studies to evaluate the matrix interference. Known amounts of NRS were spiked into both the buffer and placebo matrices, and recovery was calculated as follows:

Spiked Recovery (%) = [(C_spiked – C_unspiked) / C_added] × 100

This approach allowed for the identification of potential interference due to sample matrix components. Recovery values within 80–120% were considered acceptable in accordance with the ICH Q2(R1) guidelines [19].

### Recovery and Repeatability Over the Concentration Range

Recovery and repeatability were assessed using serially diluted NRS samples at various concentrations. Each dilution was analyzed in replicates, and consistency was confirmed by evaluating the recovery and CV across replicates. Recovery (%) was calculated using the following equation:

Recovery (%) = ((Measured Value) / (Expected Value)) × 100

Additionally, human TIG biological reference preparation Batch 2 (code: H1110000) was used to prepare standard dilutions and to verify the assay range according to the USP <1033> guidelines.

To validate ELISA results, human TIG biological reference preparation Batch 2 (code: H1110000) was obtained from the Korean Ministry of Food and Drug Safety. A series of dilution standards was prepared to evaluate the correlation and determine the relative potency, establishing the assay range in accordance with the United States Pharmacopeia (USP) <1033> guidelines for biological assay validation.

### ELISA Suitability Evaluation

A validated ELISA developed to assess the potency of anti-TIG was used to evaluate its suitability in comparison with various commercially available kits. The ELISA Classic Tetanus IgG Test (SERION, ESR108G, Germany) and Anti-Tetanus Toxoid IgG EIA Binding Site, MK010.U, United Kingdom) were used to analyze GM, GCV, *p*-value, and recovery.

## Results

### Risk Assessment of ELISA

For the development of an ELISA based on USP <1220>, ICH Q14, and Q2(R2), factors influencing the assay were systematically analyzed and visualized using an Ishikawa diagram (Fig. S1). Additionally, the FMEA table was constructed to prioritize individual influencing factors (Table 1). Each factor was assigned a score from 1 to 5 based on its impact on ATP accuracy, precision, and specificity. For example, the number of washing steps in the ELISA procedure had a minor effect on accuracy (score = 2, priority = 10), a significant influence on recovery (score = 3, priority = 8), and a definite impact on precision (score = 4, priority = 8). This yielded a total risk value of 76. Based on the risk scoring of the influencing factors, those with total scores exceeding 50 were considered to have a greater impact on the assay and were selected as analytical procedure parameters. These critical factors, initially identified through the Ishikawa diagram and prioritized by FMEA, were directly selected as key input variables for the subsequent DoE. In particular, parameters with total risk scores greater than 50 in the FMEA (Table 1), including antibody incubation time, incubation temperature, color development time, and washing steps, were incorporated as variables in the DoE shown in Table 2.

### Multiple Response Optimization

**Fig. 1:**
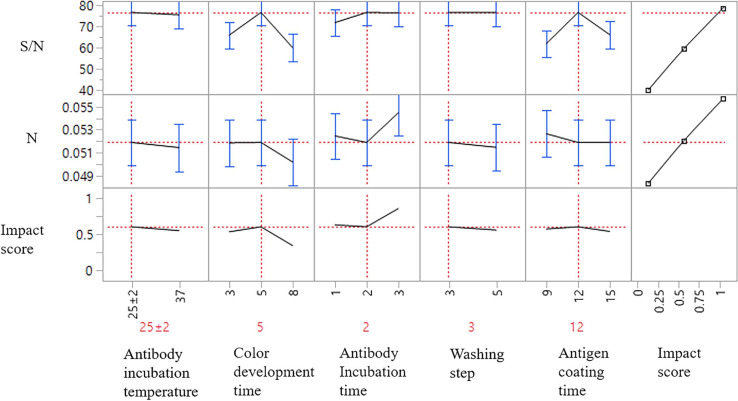
Predictive profiler of optimized assay conditions. Profile plots show the effect of individual parameters—incubation temperature, color development time, antibody incubation time, washing step, and antigen coating time—on the S/N ratio. Vertical and horizontal red lines represent the optimal settings and the corresponding predicted responses, respectively. The impact score represents a summary measure automatically derived from JMP-based multiple response optimization and reflects the overall influence of each parameter on assay performance. S/N, signal-to-noise.

Based on the results of FMEA, five critical factors influencing ELISA performance were identified. To optimize these critical factors, a total of 18 experiments were designed using JMP 18^®^ software (Table 2). The antibody incubation time was screened for 1–3 h, and antigen incubation was screened for 9–15 h. The incubation temperatures were 25 ± 2°C and 37°C. Additionally, the color development time was screened at 3, 5, and 8 min, and the washing steps were tested after three and five washes. These experimental ranges were established based on preliminary scouting experiments and further refined through a structured risk assessment: factors influencing ELISA performance were first mapped using a Ishikawa diagram and subsequently evaluated via FMEA, and high-risk parameters and their plausible operating ranges were selected as variables in the DoE design. The optimized MODR conditions that yielded the highest S/N were a 2 h antibody incubation time, 5 min color development time, 25 ± 2°C incubation temperature, three washing steps, and 12 h antigen coating time (Fig. 1). All subsequent experiments were conducted under these optimized conditions.

### Optimization of Antigen and Antibody Concentration in ELISA

**Fig. 2:**
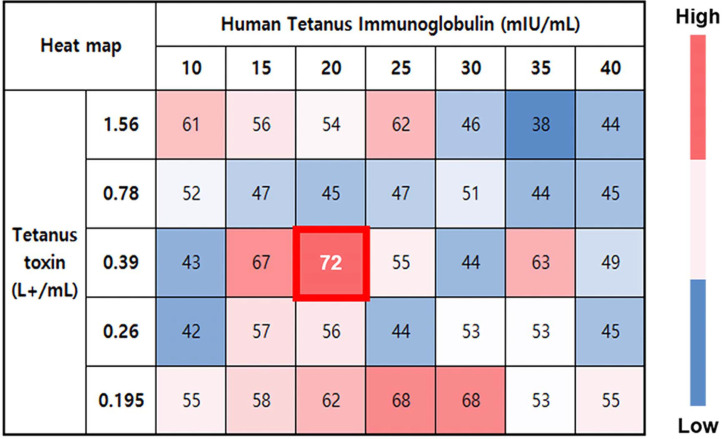
Heat map of fixed factors. Heat map showing eight color grades of S/N values (38–71) across combinations of tetanus toxin concentrations (L+/mL) and human tetanus immunoglobulin concentrations (mIU/mL). The condition yielding the maximum S/N ratio is indicated. S/N, signal-to-noise.

A dose–response analysis was performed by serially diluting the tetanus toxin and NRS, and the linear range was identified using a 4PL regression curve (data not shown). The response curves enabled the identification of a concentration window that produced consistent and proportional OD values relative to the analyte levels. Within this range, the highest S/N ratio was observed at 0.39 L+/mL tetanus toxin and 20 mIU/mL NRS, which were selected as the optimal antigen and antibody concentrations for the assay (Fig. 2).

### Validation of Method: Linearity, Accuracy, and Precision

The optimized ELISA method was validated for linearity, accuracy, precision, and recovery in accordance with ICH Q2(R1) and USP <1033> guidelines.

**Fig. 3:**
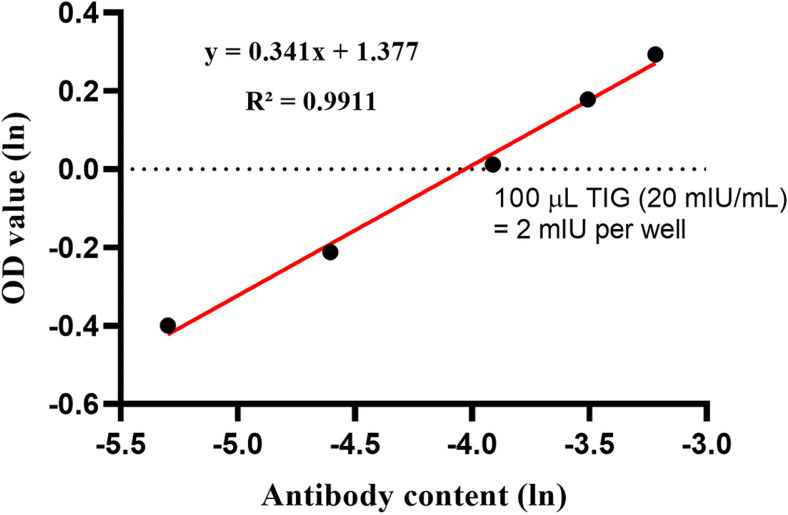
ELISA linearity validation of NRS. Linearity was confirmed through three independent experiments. The figure presents the mean values obtained from repeated measurements.

Linearity was evaluated using serial dilutions of the NRS, and logarithmic OD values were plotted against the natural log-transformed antibody concentrations. The resulting curve exhibited excellent linearity, with a coefficient of determination (R^2^ = 0.9911; Fig. 3), confirming the ability of the assay to provide consistent responses across a range of concentrations.

Accuracy and precision were evaluated under intermediate precision conditions. Two analysts conducted the assay twice daily for two consecutive days. The accuracy was determined as the percentage bias and its confidence interval, and the precision was evaluated using GM, CV, and GCV. All values were within the predefined acceptance criteria (Table 3).

### Specificity Assessment

Specificity was evaluated in accordance with the ICH Q2(R1) guideline to verify that the ELISA accurately detected TIG in the presence of matrix components. Placebo samples lacking the active substance were tested for nonspecific binding, and TIG was spiked into both the buffer and placebo matrices. The spiked recovery was calculated to determine whether the matrix components interfered with analyte detection. As shown in Table 4, the spiked quantities ranged from 3.5 to 17.5 mIU/mL, and all recovery values fell within the acceptable range of 80–120%, confirming the selective detection and absence of matrix interference. These results demonstrate that this method can selectively detect TIG without interference from formulation excipients or placebo matrices.

### Recovery and Repeatability Across Concentration Range

To confirm the consistency and reproducibility of the method across the working range, the recovery and repeatability were evaluated at six concentration levels using 25 replicate measurements per level. As summarized in Table 5, all recovery values were within the acceptance criteria (80–120%), and the CV values remained low across all tested concentrations. These results demonstrate that the ELISA method provides reliable and repeatable performance across a wide range of TIG concentrations, supporting its applicability for *in vitro* potency assessments in routine quality control.

### ATP of ELISA Method

The ATP was defined to ensure that the developed method met its intended purpose of accurately, precisely, and specifically measuring human TIG potency. The ATP included criteria for linearity (R^2^ ≥ 0.99), accuracy (bias within ±15%), precision (GCV ≤ 20%), and recovery (within 80–120%). These thresholds were established in accordance with ICH Q2(R1) and USP <1033> guidelines. All the validation results met the ATP specifications, supporting the suitability of the method for its intended use (Table 6).

### Comparison with a Commercial ELISA Kit

To assess the applicability of the developed ELISA method, a comparative analysis was performed using two commercially available TIG ELISA kits: one from The Binding Site (UK) and the other from Serion-Virion (Germany). GM, GCV, and recovery were evaluated for each method. The results indicated no substantial differences in GM and GCV values, and the recovery rates were consistent across all platforms. Statistical analysis was performed using a one-way ANOVA, based on data obtained from at least three independent replicates (n ≥ 3) for each method. No statistically significant differences were observed between the developed ELISA method and the commercial kits (*P* = 0.6108). Statistical analysis showed a P-value of 0.6108, indicating no significant difference between the developed method and commercial kits. These findings confirmed that the validated ELISA provides comparable accuracy and precision, supporting its practical use along with existing commercial assays (Table 7).

## Discussion

Our study aimed to develop and validate a robust, animal-free ELISA method for assessing the *in vitro* potency of human TIG using AQbD principles. This approach provides a structured, risk-based framework for analytical development that improves both scientific robustness and regulatory alignment [20, 21]. Compared with previously reported ELISA or AQbD-based methods, our work distinguishes itself by applying a comprehensive AQbD framework to a regulatory-grade *in vitro* potency assay for human TIG, with explicit alignment to ICH and USP requirements. This positions the assay not only as an ethical alternative to TNT but also as a method that can be directly integrated into real-world quality control workflows for plasma-derived immunoglobulin products.

Reliable and animal-free alternatives to traditional *in vivo* potency assays are urgently needed to meet both scientific and regulatory demands for biological quality control [5, 21]. To address this need, we applied AQbD principles to develop and validate an ELISA method for evaluating the *in vitro* potency of human TIG. Conventional ELISA optimization often lacks a systematic approach to identifying variables that critically influence assay performance, so methods tend to rely on trial-and-error and may yield inconsistent analytical outcomes [22]. By contrast, the AQbD-based framework enabled systematic identification and prioritization of critical parameters, leading to more consistent and reliable results.

Furthermore, ELISA optimization is often limited by one-factor-at-a-time experiments, which fail to capture interactions between variables. To address this limitation, we applied a DoE-based multivariate optimization strategy [23]. This approach enabled the simultaneous evaluation of multiple parameters and their interactions, leading to improved assay sensitivity and reduced variability.

In many ELISA-based potency assays, the antigen and antibody concentrations are selected empirically without quantitative justification, which can lead to reduced sensitivity and suboptimal assay responsiveness [24]. To overcome this limitation, we systematically optimized both antigen and antibody concentrations using dose–response experiments based on the NRS. Tetanus toxin and NRS were serially diluted across a defined range, and the data were modeled using 4PL regression to determine the optimal concentration range. The assay achieved the highest S/N ratio of 72 at 20 mIU/mL NRS and 0.39 L+/mL tetanus toxin. In addition, the assay demonstrated excellent linearity within a range of 10.23–32.74 mIU/mL, providing a reliable foundation for accurate potency quantification.

To ensure regulatory compliance, ELISA methods must meet the ICH and USP analytical performance criteria. Many in-house assays fail to satisfy these standards owing to insufficient systematic validation. To address this issue, we validated the optimized method following the ICH Q2(R1) and USP <1033> guidelines. The assay demonstrated excellent linearity (R^2^ > 0.99), accuracy (GM > 99%), and precision (low GCV%), with recovery values consistently within the 80–120% acceptance range. The total analytical error was 4.9%, which is well within the ATP-defined threshold, confirming the robustness of the method.

Finally, newly developed methods must be benchmarked against established commercial platforms to confirm their practical applicability. Although several alternative methods are promising, they often lack comparative validation. We compared the performance of our method with those of two commercial ELISA kits and found no statistically significant differences in GM, GCV, or recovery (*P* = 0.6108). These results demonstrate that the AQbD-based ELISA performs equivalently to existing commercial assays in terms of accuracy and reproducibility.

Beyond analytical validation, the developed ELISA method has direct practical relevance as a candidate routine potency test for human TIG, offering an animal-free alternative to TNT that can be implemented in quality control laboratories for lot release and stability testing. Compared with the traditional animal-based TNT, the proposed method shortens assay turnaround time and reduces inter-assay variability, thereby improving efficiency and reproducibility in quality control workflows. Furthermore, this AQbD-based approach may be extended to other plasma-derived immunoglobulin products as an *in vitro* potency assay platform, although additional studies are required to confirm its applicability across different IgG formulations.

Despite these strengths, several limitations should be acknowledged. First, the assay was evaluated using a limited set of human TIG preparations, and its applicability to other plasma-derived immunoglobulin products has not yet been fully established. Second, all experiments were performed within a single laboratory; therefore, potential inter-laboratory variability and transferability to other settings remain to be demonstrated. Finally, long-term robustness, including performance under routine use over extended periods and potential impacts of reagent or operator changes, will require further investigation in future studies. Although detailed TNT data generated in a regulatory setting cannot be disclosed in this manuscript because of their confidential nature, the available experience indicates that the ELISA provides lot-release decisions that are concordant with those of the traditional TNT.

Overall, the validated method presents a scientifically sound, regulatory-aligned, and ethically favorable alternative to the traditional TNT, which is labor-intensive, variable, and ethically concerning owing to its animal use [5]. By aligning with the 3Rs principle and current global regulatory expectations, this study contributes to the advancement of modern analytical strategies for vaccine and biological quality control.

## Conclusion

This study successfully developed and validated an animal-free ELISA method for the *in vitro* potency assessment of human tetanus immunoglobulin, utilizing AQbD principles. By integrating systematic risk assessment, multivariate optimization through DoE, and rigorous validation aligned with ICH Q2(R1) and USP <1033> guidelines, the method demonstrated excellent linearity, accuracy, precision, and specificity. Comparative analysis confirmed that the assay performs equivalently to commercial ELISA kits, further supporting its applicability for routine use. This robust and reproducible assay provides a scientifically sound and ethically favorable alternative to traditional *in vivo* testing methods, advancing the 3Rs principles and meeting modern regulatory expectations for biologics quality control.

## Supplemental Materials

Supplementary data for this paper are available on-line only at http://jmb.or.kr.



## Figures and Tables

**Table 1:** Failure mode effect analysis (FMEA) of critical ELISA parameters.

Rating of Importance (Priority)			10	8	8	
ID	Process Step	Process Input	Accuracy	Specificity	Precision	Total
1	96-well Plate	Brand	1	1	1	26
2	Blocking	Buffer	1	2	1	34
3	Secondary Antibody	Dilution	1	1	2	34
4	Sample Dilution	Tip, vials	1	1	2	34
5	Color Development	Temperature	1	2	2	42
6	Antigen Incubation	Temperature	2	1	2	44
7	Washing	Volume	2	2	2	52
8	Sample Dilution	Humidity	3	1	2	54
9	Substrate Reaction	Temperature	2	2	3	60
10	Sample Dilution	Pipetting technique	2	3	3	68
11	Washing	Step	2	3	4	76
12	Antibody Incubation	Time	3	3	4	86
13	Substrate Reaction	Time	4	3	4	96
14	Antibody Incubation	Temperature	4	4	5	112
15	Antigen Incubation	Time	5	5	5	130

**Table 2:** DoE-based optimization of critical ELISA parameters.

Test	Antibody incubation time (h)	Incubation temperature (°C)	Color development Time (min)	Washing Time (step)	Antibody Incubation Time (h)	S/N	N
1	1	25 ± 2	3	5	9	45	0.053
2	3	37	3	5	15	55	0.054
3	2	25 ± 2	5	5	12	77	0.051
4	2	37	8	5	9	46	0.046
5	3	37	5	3	9	67	0.055
6	3	25 ± 2	8	5	15	50	0.052
7	1	25 ± 2	5	5	15	63	0.052
8	3	25 ± 2	3	3	12	63	0.055
9	2	25 ± 2	8	3	9	43	0.053
10	3	37	5	5	9	53	0.055
11	2	25 ± 2	5	3	15	65	0.052
12	1	37	5	3	12	70	0.052
13	3	25 ± 2	8	3	12	60	0.051
14	2	37	3	5	12	67	0.050
15	2	37	3	3	15	52	0.051
16	1	37	8	5	12	53	0.051
17	1	37	8	3	15	42	0.050
18	1	25 ± 2	1	3	9	48	0.052

S/N: Signal to noise ratio, N: Noise

**Table 3:** Accuracy and precision of the developed ELISA method.

Antibody Content (IU/mL)	0.04	0.03	0.25	0.02	0.01	0.005
Bias (%)	2.26	-3.12	-9.86	-5.2	0.98	5.64
Confidence interval for bias (%)	-2.30 to -1.4	0.45- to -1.39	-1.85 to -0.72	-0.23 to -0.80	-0.50 to 0.40	0.41 to -0.13
Geometric mean (%)	101.92	96.27	89.13	94.31	100.52	105.45
Geometric coefficient of variation (%)	8.28	11.5	7.95	10.39	9.82	6.11

**Table 4:** Specificity assessment via spiked recovery.

	Spiked Quantity (mIU/mL)	Spiked Recovery (%)
EDQM	17.5	87.39
	8.5	110.32
	6.5	108.82
	5	113.93
	4	91.23
	3.5	103.98

**Table 5:** Recovery and repeatability of ELISA across concentration levels.

Antibody Content (IU/mL)	Recovery (%)	Repeatability (%)
0.4	102.26	8.30
0.3	96.88	9.68
0.25	89.40	8.13
0.2	94.80	9.85
0.1	100.98	9.53
0.05	105.64	6.19

**Table 6:** Comparison of final method performance with ATP.

	Development Objectives	Development Results
Intended purpose	Development of an ELISA Method for HTIG Using the NRS	
Linearity	R^2^ ≥ 0.95	R^2^ ≥ 0.99
Accuracy	|Bias| ≤ 15%	|Bias| ≤ 9.86%
Precision	≥ 85%	89.13–105.45%
Specificity	80–120%	87.39–113.93%
Recovery	80–120%	89.4–105.64%
Repeatability	CV ≤ 10%	6.19–9.85%
Specification range to be controlled by this method	5–40 mIU	3.5–40 mIU

Note: Column (A) represents the analytical target prior to the establishment of the method. Upon completion of method development, column (B) was designed based on validation results to incorporate the optimized analytical conditions

**Table 7:** Comparative evaluation of the developed ELISA NG method and commercial kits.

	Manual	Serion	The Binding Site
Geometric mean (%)	97.5	82.7	104.1
Geometric coefficient of variation (%)	1	1.2	1
Recovery (%)	102.7	99.1	102.6

Note: Recovery values are mean ± SD (n ≥ 3). GCV, geometric coefficient of variation.
