# Supplementary material for: Analytical Quality-by-Design-Based Development of an ELISA for In Vitro Assessment of Human Tetanus Immunoglobulin Potency
Source: J Microbiol Biotechnol. 2026 Jul 1;36:e2603028. doi: 10.4014/jmb.2603.03028 (PMC13364764; doi:10.4014/jmb.2603.03028)
Supplement: Supplementary file 1 [file jmb-36-e2603028-supple.pdf]

J. Microbiol. Biotechnol. 2026. 36: e2603028
https://doi.org/10.4014/jmb.2603.03028

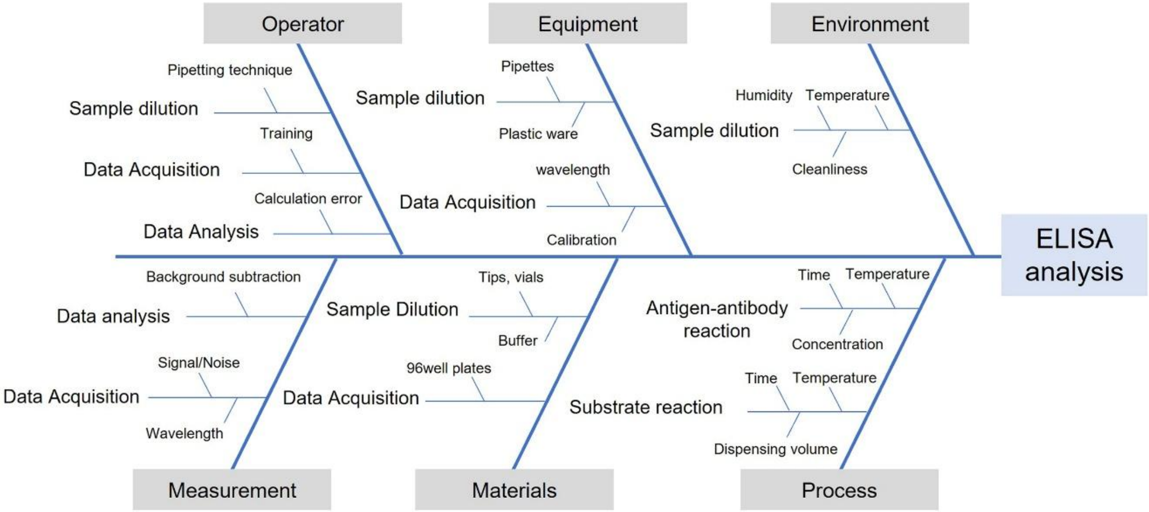

Fig. S1. Ishikawa diagram of ELISA assay variability factors.

Potential sources of assay variability were categorized into six major groups—operator, equipment, environment, measurement, materials, and process—to systematically identify critical factors that may influence ELISA performance.
